# Lipid Membranes in Poxvirus Replication

**DOI:** 10.3390/v2040972

**Published:** 2010-04-06

**Authors:** Jason P. Laliberte, Bernard Moss

**Affiliations:** Laboratory of Viral Diseases, National Institute of Allergy and Infectious Diseases, National Institutes of Health, Bethesda, MD 20892, USA; E-Mail: lalibertej@niaid.nih.gov

**Keywords:** phospholipids, transmembrane proteins, virus entry, endocytosis, virus assembly, exocytosis

## Abstract

Poxviruses replicate in the cytoplasm, where they acquire multiple lipoprotein membranes. Although a proposal that the initial membrane arises *de novo* has not been substantiated, there is no accepted explanation for its formation from cellular membranes. A subsequent membrane-wrapping step involving modified trans-Golgi or endosomal cisternae results in a particle with three membranes. These wrapped virions traverse the cytoplasm on microtubules; the outermost membrane is lost during exocytosis, the middle one is lost just prior to cell entry, and the remaining membrane fuses with the cell to allow the virus core to enter the cytoplasm and initiate a new infection.

## 1. Introduction

The *Poxviridae* is a family of large, complex, enveloped DNA viruses that infect a variety of vertebrate and invertebrate hosts. Poxviruses are of significance both medically and scientifically due to their wide distribution, pathogenicity, and unique cytoplasmic replicative life cycle [1]. Several prominent members, including variola virus (causative agent of smallpox), molluscum contagiosum virus (cause of a common skin infection of young children and immunosuppressed adults) and monkeypox virus (agent of a smallpox-like disease in parts of Africa), are of considerable concern for public health and biodefense. The prototypic and most studied poxvirus – vaccinia virus (VACV) – serves as an effective smallpox vaccine, a platform for recombinant vaccines against other pathogens and an efficient gene expression vector for basic research [2–4].

Along its approximate 195-kbp double-stranded DNA genome, VACV encodes ∼200 proteins, ranging in function from viral RNA and DNA synthesis and virion assembly to modulation of host immune defenses [1]. The most abundant and simplest infectious form of the poxvirus particle – the mature virion (MV; alternate name: intracellular mature virion [IMV]) – consists of the viral DNA genome encased in a proteinaceous core and an outer lipoprotein membrane with ∼60 and ∼25 associated viral proteins, respectively [5]. The presence of an inner membrane forming one layer of the core wall has been suggested by cryo-electron tomography [6] but not yet verified by other methods.

Following attachment to cell surfaces and fusion with the plasma or endosomal membrane, poxvirus replication is initiated by entry of the viral core into the cytoplasm where all subsequent steps of the life cycle take place [7]. Poxvirus cores harbor the viral DNA-dependent RNA polymerase and transcription factors necessary for expression of early genes, which constitute nearly half of the viral genome and encode proteins needed for DNA replication and intermediate gene transcription as well as a large number of immunomodulators [1]. Poxviruses exhibit a temporally-regulated gene expression program, *i.e.*, expression of early genes encoding DNA replication and intermediate transcription factors triggers the expression of intermediate genes encoding late gene specific transcription factors [8]. Late gene products primarily consist of structural proteins needed for progeny virion assembly as well as those enzymes destined for incorporation into progeny virions and used for early gene expression during the next round of infection.

Assembly of the MV involves more than 80 viral gene products [5]. In addition, during transit through the cytoplasm, a subset of progeny MVs acquires two additional membrane bilayers, one of which is lost during exocytosis of the particle, to yield the less abundant enveloped virion (EV; alternate names: cell-associated enveloped virion [CEV] and extracellular enveloped virion [EEV]). Thus, an EV is essentially an MV with an additional membrane in which at least six unique proteins are associated [9]. Of note, EVs are antigenically distinct from MVs [10] and are important for efficient virus dissemination in the infected host and protection against immune defenses [9]. In contrast, MVs are released upon cell lysis and may be important for animal-to-animal transmission.

This review highlights the role of lipoprotein membranes in poxvirus entry into cells and during the assembly, morphogenesis and egress of progeny virions.

## 2. Review

### 2.1. Roles of membranes and lipids in cell entry

#### 2.1.1. Entry of MVs

**Figure 1.**
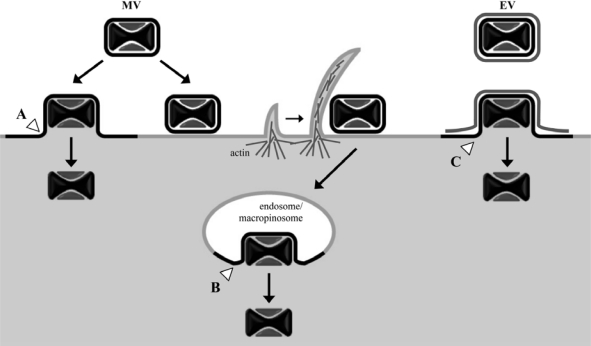
Cell entry pathways of vaccinia virus mature virions (*MV*) and enveloped virions (*EV*) following attachment at the cell plasma membrane. Depending on the VACV strain and target cell, MV cores can enter the cytosol either through direct fusion of the MV membrane with the plasma membrane (*A*) or, following endocytosis/macropinocytosis, through fusion of the MV membrane with vesicles (*B*). EV particles have been observed to shed their additional membrane bilayer prior to direct fusion of the underlying MV membrane with the cell plasma membrane (*C*); entry of EV through an endosomal route might also occur. White arrowheads denote sites of membrane fusion between the MV particle membrane and cellular membranes.

MVs have been subjected to the great majority of VACV entry studies primarily because they can be highly purified, are stable and are the simplest infectious particle. In addition, the MV is the form used for vaccination. The initial attachment of MVs to cells occurs, in part, through interactions between viral membrane proteins (D8, A27, H3, and A26) and cell surface glycosaminoglycans (specifically, chondroitin sulfate and heparan sulfate) or laminin; however, individually these proteins are not essential [11–14]. Viral-cell membrane fusion and entry of the virus core into the cytoplasm can occur at neutral pH at the cell surface, as evidenced by numerous electron micrographs and suggested by biochemical membrane fusion assays [15–19]. Electron micrographs have also visualized the uptake of MVs into vesicles [20]. These observations were reconciled in a recent study showing that both fusion at the plasma membrane and endosomal vesicles can occur even in the same cell [21] (Figure 1). Further studies showed that the predominant mechanism of entry of the WR strain of VACV is by a low pH-dependent mechanism [21] following macropinocytosis [22,23]. The situation remains complicated, however, as there are VACV strain differences regarding the relative contributions of low and neutral pH entry mechanisms [24].

In terms of the number of proteins required, VACV membrane fusion is far more complex than any other studied viral system. At least twelve MV membrane proteins (A16, A21, A28, G3, G9, H2, J5, L5, O3, I2, L1 and F9) – nine or ten of which function in a multi-protein complex termed the entry/fusion complex (EFC) – are individually required [25–35]. Two EFC proteins (A16 and G9) and the EFC-associated L1 protein are myristoylated, but the significance of these lipid moieties in virus entry has not been determined.

#### 2.1.2. Lipids of the MV membrane

Lipids, mostly phospholipids and cholesterol, constitute approximately 5% of the MV mass [36]. The MV contains several phospholipids with an abundance of phosphatidylcholine and lesser amounts of phosphatidylethanolamine, phosphatidylinositol, and phosphatidylserine (PS) [37–39]. An additional phospholipid, semilysobisphosphatidic acid (also known as acylbis(monoacylglycero)phosphate or acylphosphatidylglycerol) has been identified in MVs [39,40]. Quantitation of the lipids in the MV by Cluett and Machamer [39] is shown in Table 1. The lipid composition likely reflects the cellular origin of the MV membrane.

Early studies by Ichihashi and Oie demonstrated the importance of virion-incorporated PS for VACV MV infectivity [41,42]. Extraction of virion-associated lipids with Nonidet P-40 detergent rendered MVs non-infectious, but incubation with either crude uninfected cell membrane preparations or PS-containing liposomes partially restored infectivity [41,42]. More recently, these results were extended and an apoptotic mimicry model for poxvirus entry, in which virion-associated PS specifically served to flag virions as apoptotic debris for cell uptake by macropinocytosis, was proposed [23]. However, PS is not unique in its ability to trigger this uptake mechanism as the reconstitution of detergent-extracted MVs with several other anionic phospholipid species could also facilitate virus entry in the absence of PS [43]. Nonetheless, these studies indicated the importance of the MV membrane lipid composition, specifically the presence of anionic phospholipid species, in cell entry of VACV MVs.

#### 2.1.3. Entry of EVs and the EV-specific membrane

The EV contains an additional outer membrane compared to the MV. Relative to MVs, EVs were reported to be enriched in sphingomyelin and PS with lower amounts of phosphatidylinositol [38]. In a later section, we will discuss the origin and phospholipid composition of the wrapped virion, which contains an additional membrane relative to the EV. MVs and EVs bind differently to cells [44] but little is known regarding the mechanism or proteins required for binding of the latter particles. Entry of EVs necessitates rupture of the outer membrane to expose the EFC embedded in the MV membrane [7] (Figure 1). Electron micrographs reveal a clear dissociation of the EV membrane “wrapper” from individual particles at the cell surface; importantly, no continuity of the EV wrapper with the plasma membrane was observed confirming that it is not fusogenic [45]. This “ligand-dependent non-fusogenic dissolution” of the outer EV membrane requires the EV membrane glycoproteins A34 and B5 and interactions with cell surface polyanionic molecules, e.g., glycosoaminoglycans, following virus attachment [45,46]. The subsequent entry steps are reported to be low pH-dependent [47].

#### 2.1.4. Lipids and protein components of the target cell membrane

Cell surface glycosaminoglycans and laminin are important for the initial attachment of MVs to the cell surface [11,13,14,48]. The binding of MVs to cells induces the formation of actin-enriched membrane protrusions that engulf the virus particles for entry by macropinocytosis; this cellular response involves actin rearrangements and Rac1 GTPase as well as several serine/threonine, tyrosine, and phosphatidylinositol kinases including Pak1, PKC, and PI(3)K [23,49]. In contrast to MVs, entry of EVs does not seem to be signaling dependent [49]. Specific cell receptors, for either the MV or EV forms of VACV, have not been identified. Although PS-specific receptors for MV have been suggested [23], this is unlikely since a subsequent study indicated that PS is not uniquely involved in entry [43]. Depletion of cellular cholesterol levels does not affect cell binding of MVs but significantly inhibits entry of viral cores [50] indicating the importance of the composition and organization of the plasma membrane and suggesting a role for lipid rafts. Indeed, the block in MV penetration of cholesterol-depleted cells was found to occur at the initial stages of virus-cell membrane fusion (Laliberte and Moss, unpublished). Although the precise role of cellular cholesterol in VACV membrane fusion is not known, it may be indirectly required for triggering specific cell signaling pathways by cell surface-bound MVs. Additionally, reduced levels of cholesterol in the target cell membrane may affect cell surface distribution of an unidentified cell receptor(s) or other factor(s) required for virus entry.

### 2.2. Role of membranes and lipids during morphogenesis and egress of progeny virions

#### 2.2.1. Membranes associated with virus factories and immature virions (IVs)

After virus-cell membrane fusion, viral cores are actively transported on microtubules away from the cell periphery and deeper into the cytoplasm [51], where early gene transcription occurs. DNA from a single core may serve as a template for genome replication forming a viral factory [52–54]. Remarkably, at an early stage, each individual factory is entirely surrounded by ER membranes [55]. It has been suggested that the latter membranes are associated with replicating DNA. Following viral DNA replication and subsequent intermediate gene expression, the accumulation of late gene products – including viral structural proteins and enzymes/early gene transcription factors destined for virus packaging – allows poxvirus assembly and morphogenesis. The highly complex process of generating progeny virions is orchestrated by more than 80 viral gene products [5].

**Figure 2.**
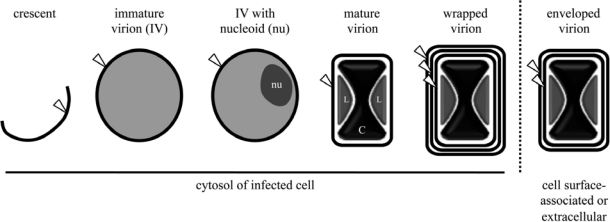
Enumeration of lipid membrane bilayers (white arrowhead) possessed by vaccinia virus assembly intermediates. Nucleoids (*nu*) are electron-dense, DNA-containing substructures of immature virions. Lateral bodies (*L*) are internal virion structures of heterogenous material surrounding the dumbbell-shaped viral core (*C*). The location of each morphogenic form is indicated below the respective schematic.

Viral membranes play a pivotal role in virion assembly. The initial structure observed by electron microscopy is the viral crescent membrane, which expands to form the spherical immature virion (IV) (Figure 2). The number of lipid bilayers comprising the viral crescent and IV membranes has been the subject of controversy. Electron microscopic images from several different laboratories were consistently interpreted as showing a single bilayer with an external spicule border [56–61]. Nevertheless, others interpreted their images as demonstrating two closely apposed bilayers [38,62,63]. The controversy appears to have been settled as no evidence for two bilayers was obtained by freeze-fracture studies [64] and the initial proponents of the double bilayer now also favor a single bilayer [65]. The spicule border on the crescent and IV membrane has been shown to be a honeycomb lattice scaffold composed of trimers of the viral D13 protein [64,66]; it is possible that the separation of this protein scaffold from the underlying membrane provided images suggesting a double membrane bilayer.

Neither the crescent nor the IV membrane has been extensively purified and characterized so their lipid compositions remain unknown. The composition is usually inferred to be that of the MV membrane. A number of viral protein components of the crescent and IV membrane have been determined by immunoelectron microscopy and will be discussed below.

#### 2.2.2. Assembly of the IV membrane

The unusual appearance of the crescent and IV membranes in cytoplasmic virus factories and their separation from cellular organelles led to the idea of their *de novo* synthesis [56]. However, the unprecedented nature of spontaneous membrane formation encouraged efforts to find a cellular organelle from which the viral membrane might be derived. Electron microscopic images have depicted close association of membranes of the ER-Golgi apparatus intermediate compartment (ERGIC) with IVs [38,63,67,68] while others have shown a close association with ER membranes [69]. Nevertheless, direct continuity between cellular organelles and viral membranes has not been demonstrated unambiguously. Although viral membrane proteins have been detected by immunogold labeling in ERGIC and ER, the reverse has not been shown, *i.e.*, cellular proteins in crescent or IV membranes. ERGIC resident proteins have also not been detected in purified MV particles, the membrane of which is presumably derived from the IV [70]. The lipid profile of purified MV particles was interpreted as being consistent with the MV membrane being of intermediate compartment or *cis*-Golgi network origin [39]. However, inhibiting transport of proteins from the ER to the Golgi compartment does not prevent the formation of IVs and MVs [71], implying that IV membranes are not derived from the ERGIC or Golgi compartment. Additional studies have shown that viral proteins can transit directly through the ER to the IV and accumulate in the MV membrane [69].

In addition to the viral D13 scaffold protein, several membrane (A14 and A17) [72] and non-membrane (A11, F10, G5, and H5) [73–77] viral proteins are required for crescent formation, whereas other proteins such as A9 [78] associate with the crescent membrane but are required at a later stage of morphogenesis. The exact roles of A14 and A17 proteins in crescent formation are uncertain but in the absence of either membrane protein crescent formation is reduced or aberrant and small vesicles accumulate [79–82].

Elongation and closure of viral crescents around electron dense viroplasm, containing core proteins or their precursors, give rise to the spherical IVs, which are approximately 350 nm in diameter [5] (Figure 2). Association of viroplasm with crescents is dependent on the expression of a complex of seven viral core proteins (A15, A30, D2, D3, F10, G7, and J1) [83]. Following packaging of the viral genome, the IV particle undergoes a gross alteration to become a barrel-shaped particle distinctive of poxvirus MVs. This transition is accompanied by disassembly of the viral D13 scaffold [84] and the processing of core protein precursors [85,86]. Virion assembly has recently been reviewed in detail [5]. Although crucial for progeny virion infectivity, the EFC as well as the EFC-associated proteins L1 and F9 are dispensable for the MV morphogenesis.

#### 2.2.3. Membranes of the enveloped particle

As described earlier, after completion of MV morphogenesis, some particles are actively transported along microtubules away from the viral factory to sites where wrapping [87,88] with modified cisternae of endosomal [89] or trans-Golgi [90,91] origin occurs. These wrapped virions (WV; alternate name: intracellular enveloped virion [IEV]) possess an MV membrane and two additional membrane bilayers (Figure 2). The phospholipids of the WV have been analyzed and the composition of the wrapping membrane inferred by subtracting the values for the MV from the WV [39] (Table 1). The phospholipid components of the WV, with the exception of *N*-acyl phosphatidylethanolamine, are present in 2 to 3 times the molar amount of the MV, consistent with additional lipid bilayers in the WV (Table 1). At least seven proteins are associated with the wrapping membrane: A33, A34, A36, A56, B5, F12, F13. Of these, A36 and F12 are located exclusively on the outermost WV membrane. Although the details by which MVs acquire these membranes are unclear, the process requires the expression of several viral proteins, including the MV membrane-associated A27 protein [88,92] and the WV membrane-associated B5 [93,94] and F13 proteins [95]; other WV associated viral proteins appear necessary for efficient MV particle wrapping (reviewed in [9]). Of note, the peripheral F13 membrane protein is palmitylated on a pair of cysteine residues and addition of these 16-carbon fatty acid tails is necessary for membrane association, proper localization, and function of F13 [90,96,97]. The importance of the F13 protein is consistent with the potency both *in vitro* and *in vivo* of the anti-poxvirus therapeutic ST-246, which inhibits F13 protein activity in the infected cell [98].

Wrapped virions are transported to the cell periphery in a microtubule-dependent manner relying on an interaction between the motor protein kinesin-1 and the viral A36 protein [99] and the viral F12 protein [100]. Once near the plasma membrane, the WV particle traverses the cortical membrane cytoskeleton by enhancing microtubule dynamics near the cell surface and inducing reorganization of the actin cytoskeleton resulting from the inhibition of RhoA signaling by the viral F11 protein [101,102]. Following dissociation from microtubules, the outermost membrane bilayer fuses with the plasma membrane resulting in the exocytosis of a doubly-wrapped EV at the cell surface. It is likely that cellular mechanisms are used for this exocytosis step since no viral mutants that have a phenotype in which virions collect under the cell surface have been isolated. Most of the exocytosed particles remain attached to the exterior of the cell and have been referred to as cell-associated EVs (CEV) to distinguish them from the released EVs (alternate name: extracellular enveloped virions [EEV]) [103]. The cell-associated EVs are largely responsible for cell-to-cell spread at the tips of actin tails.

## 3. Conclusions

Lipoprotein membranes have crucial roles at key stages of the poxvirus life cycle, namely entry, assembly, intracellular transport, exocytosis and spread. The MV membrane is comprised of phospholipids and numerous proteins that together enable attachment and fusion with either the plasma membrane or endocytic vesicles following macropinocytosis. After entry of the core into the cytoplasm, ER membranes surround the early viral factories within which DNA replication occurs. The very first recognizable viral structures in the factories are crescent membranes of yet unknown origin that engulf viroplasm, containing core proteins, to form IVs. The external lattice scaffold responsible for the spherical shape of the IV detaches from the membrane allowing further steps in morphogenesis resulting in the barrel or brick-shaped MV. The latter acquires two additional lipoprotein membranes, derived from trans-Golgi or endosomal cisternae modified by the insertion of viral proteins, that allow high speed transit of the triple-membrane layered virions on microtubules. Upon reaching the periphery of the cell, the outermost viral membrane fuses with the plasma membrane resulting in exocytosis of the double-membrane EV. Upon contacting an uninfected cell, usually at the end of a long actin-containing projection, the outer membrane is disrupted allowing fusion of the MV membrane with the cell and penetration of the viral core into the cytoplasm for continuation of the replicative cycle.

## Figures and Tables

**Table 1.** Quantitation of phospholipids in vaccinia virus MV and WV

	μg per 10^10^ virions		
Phospholipid	MV	WV	WV-MV
*N*-acyl phosphatidylethanolamine	0.38	0.31	0
Semilysobisphosphatidic acid	1.2	3.2	2
Phosphatidylethanolamine	1.7	4.5	2.8
Phosphatidylserine	0.26	1.3	1
Phosphatidylinositol	1	2.2	1.2
Phosphatidylcholine	2.8	7.8	5
Sphingomyelin	0.6	2.1	1.5

Data from Cluett and Machamer [39].
